# Synthesis and Antimicrobial Activity of *N*-Substituted-β-amino Acid Derivatives Containing 2-Hydroxyphenyl, Benzo[*b*]phenoxazine and Quinoxaline Moieties

**DOI:** 10.3390/molecules20023170

**Published:** 2015-02-13

**Authors:** Kristina Mickevičienė, Rūta Baranauskaitė, Kristina Kantminienė, Maryna Stasevych, Olena Komarovska-Porokhnyavets, Volodymyr Novikov

**Affiliations:** 1 Department of Physical and Inorganic Chemistry, Kaunas University of Technology, Kaunas 50254, Lithuania; E-Mail: kristina.mickeviciene@ktu.lt; 2 Department of Organic Chemistry, Kaunas University of Technology, Kaunas 50254, Lithuania; E-Mail: ruta.baranauskaite7@gmail.com; 3 Department of Technology of Biologically Active Substances, Pharmacy and Biotechnology, Lviv Politechnic National University, Lviv-13 79013, Ukraine; E-Mails: vnovikov@polynet.lviv.ua (M.S.); olkomarovska@gmail.com (O.K.-P.); vnovikov@polynet.lviv.ua (V.N.)

**Keywords:** heterocycles, 1,4-naphthoquinone derivatives, hydrazones, antibacterial activity, fungicides

## Abstract

3-[(2-Hydroxyphenyl)amino]butanoic and 3-[(2-hydroxy-5-methyl(chloro)phenyl)amino]butanoic acids were converted to a series of derivatives containing hydrazide, pyrrole and chloroquinoxaline moieties. The corresponding benzo[*b*]phenoxazine derivatives were synthesized by the reaction of the obtained compounds with 2,3-dichloro-1,4-naphthoquinone. Five of the synthesized compounds exhibited good antimicrobial activity against *Staphylococcus aureus* and *Mycobacterium luteum,* whereas three compounds showed significant antifungal activity against *Candida tenuis* and *Aspergillus niger*.

## 1. Introduction

The frequency of bacterial and fungal infections is an important contemporary problem due to the emerging new infectious diseases and increasing multi-drug resistance of microbial pathogens [1]. The widespread use of antibiotics has contributed to the growing infection rate since fungal infections occur after antibiotic therapy, which has the effect of killing the beneficial bacteria that normally suppress fungi. The development of new effective antifungal and antibacterial agents is strongly needed.

β-Amino acids and their derivatives are structural units of various natural compounds, such as peptides, depsipeptides, lactones, alkaloids, and antibiotics. Bacteria, cyanobacteria, fungi, and plants often incorporate β-amino acids into secondary metabolites. Many natural compounds characterized by potent biological activities are active thanks to the presence of β-amino acid substructure [2].

Benzoquinone and naphthoquinone fragments are also often incorporated into the structure of natural biologically active compounds. In most cases, the biological activity of quinones is related to their ability to accept one and/or two electrons to form the corresponding radical anion or dianion species, as well as the acid-base properties of the compounds. The variable capacity of quinone compounds to accept electrons is due to the electron-attracting (or donating) substituents at the quinone moiety, which modulate the redox properties responsible for the resulting oxidative stress [3]. Redox properties of quinones can be tuned by their substituents [4].

Different 1,4-naphthoquinone derivatives have been reported as potent anticancer [5,6], antifungal [7,8], antibacterial [9,10,11,12,13], antiviral [14,15], and antiprotozoal therapeutic agents [16], as well as cholesterol acyltransferase inhibitors [17].

Quinoxaline and its derivatives are important nitrogen-containing heterocyclic compounds possessing various biologically interesting properties with several pharmaceutical applications. The biological applications of quinoxaline compounds include antimicrobial [18,19], anti-inflammatory [20,21], antitubercular [22], anticancer [23], and antitumor agents [24,25].

Hydrazone derivatives of heteroaromatic compounds have also been reported to possess anti-inflammatory [26,27], anticancer [28], antitumor [29], antibacterial or plant-growth activity [30,31].

Herein, we report the synthesis and biological evaluation of new β-amino acid derivatives containing aromatic, heterocyclic moiety and/or naphthoquinone fragments. The structures of the synthesized compounds were unambiguously confirmed by elemental analysis, mass spectrometry, IR, ^1^H-NMR and ^13^C-NMR spectroscopy.

## 2. Results and Discussion

### 2.1. Chemistry

**Scheme 1:**
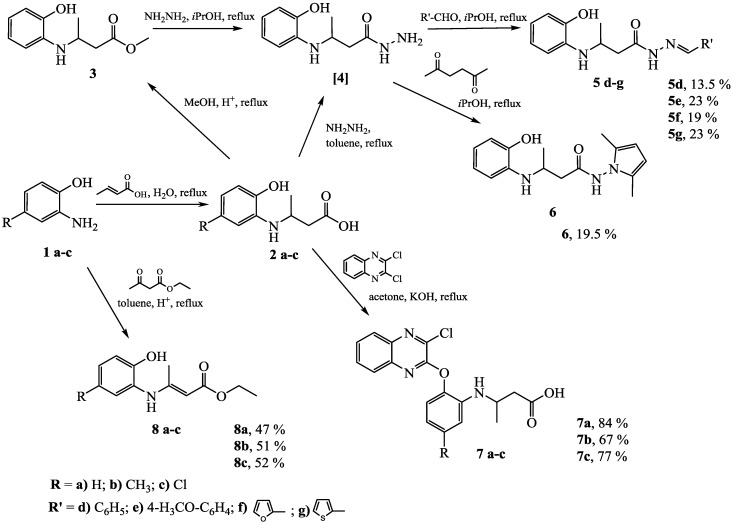
Transformation reactions of 3-[(2-hydroxyphenyl)amino]-butanoic acids **2a–c** and synthesis of ethyl-3-[(2-hydroxy-5-substitutedphenyl)amino]but-2-enoates **8a–c**.

3-[(2-Hydroxyphenyl)amino]butanoic acids **2a–c** were obtained by the reaction of the corresponding amines **1a–c** with crotonic acid (Scheme 1). The reactions were carried out under reflux in water without using organic solvents and the products were isolated by crystallization. Usually, synthesis of carboxylic acid hydrazides from esters is more facile than the one from acids. However, attempts to synthesize methyl 3-[(2-hydroxyphenyl)amino]butanoate (**3**) by esterification reaction of 3-[(2-hydroxyphenyl)amino]butanoic acid (**2a**) with methanol in the presence of a catalytic amount of sulfuric acid were unsuccessful. The target product was obtained as an oily residue and required tedious work-up procedure. Furthermore, the subsequent reaction of **3** with hydrazine hydrate gave a complex mixture of products, from which it was not possible to isolate 3-[(2-hydroxyphenyl)amino]butanehydrazide (**4**). Therefore, hydrazide **4** was prepared from **2a** by heating it under reflux with hydrazine hydrate in toluene. The residual semisolid obtained was used in subsequent reactions without further purification. The formation of ester **3** was confirmed by the presence of a singlet at 3.57 ppm attributable to the OCH_3_ group protons in the ^1^H-NMR spectrum.

Hydrazones **5d–g** were synthesized, under reflux conditions, by condensation of hydrazide **4** with different aromatic aldehydes in 2-propanol (Scheme 1). Compounds **5d–g** having an azomethine group appear as a mixture of *E* and *Z* isomers in solution [10,32,33] as it has been proven by their NMR spectra. For example, two doublets attributable to the CH_3_ group protons at 1.18 ppm (*E* isomer) and 1.22 ppm (*Z* isomer), and two doublets of doublets attributable to the CH_2_ group protons at 2.64 ppm and 3.08 ppm are observed in the ^1^H-NMR spectrum for **5d**. The NH group proton resonated as a doublet at 4.53 ppm. Protons of the CH, OH, and NH-N groups gave double sets of ^1^H-NMR resonances at 8.01 ppm (*E*) and 8.18 (*Z*), 9.22 ppm (*Z*) and 9.25 (*E*), 11.35 (*Z*) and 11.43 ppm (*E*), respectively, as well. The full set of resonances for each isomer is present in the ^13^C-NMR spectrum for **5d**. In the ^1^H- and ^13^C-NMR spectra for **5f** and **5g**, the pattern of chemical resonances indicating the formation of *E*/*Z* isomers in the solution, is analogues to the one for **5d**. The intensity ratio of the signals in all cases is 3:2 and the *Z* isomer prevails.

*N*-(2,5-Dimethyl-1*H*-pyrrol-1-yl)-3-[(2-hydroxyphenyl)amino]butanamide **6** was synthesized by the reaction of **4** with 2,5-hexanedione. The formation of pyrrole ring in compound **6** has been confirmed by the ^1^H-NMR signal at 5.62 ppm attributable to two aromatic protons.

3-{{2-[(3-Chloroquinoxalin-2-yl)oxy]phenyl}amino}butanoic acids **7a–c** were synthesized by the reaction of acids **2a–c** with 2,3-dichloroquinoxaline. The characteristic singlets attributable to the phenolic OH groups in the ^1^H-NMR spectra for compounds **2a–c** are absent in the ^1^H-NMR spectra for **7a–c**. The formation of compounds **7a–c** has been confirmed also by the increased intensity by four protons of the signals in the aromatic region.

3-(2-Hydroxyphenylamino)but-2-enoates **8a–c** were prepared by the reaction of the corresponding 2-aminophenols **1a–c** with ethyl 3-oxobutanoate. The best results were gotten when the reactions were carried out in toluene under reflux conditions. After elimination of the solvent, the products were obtained by crystallization in hexane. The formation of compounds **8a–c** has been confirmed by the presence of the resonances attributable to the protons of the =CCH_3_ group at approx. 2 ppm in the ^1^H-NMR spectra and the carbon atom signals of the methyl group at approx. 20 ppm in the ^13^C-NMR spectra. The presence of the double bond has been confirmed by the proton signal attributable to the methine group at approx. 4.5 ppm in the ^1^H-NMR spectra. In the ^13^C-NMR spectra, carbon of this group resonated at approx. 86 ppm, whereas the carbon resonance at approx. 160 ppm was attributed to the =*C*CH_3_ group. The ^1^H-NMR spectra of compounds **8a–c** display double sets of chemical resonances indicating that these compounds exist as a mixture of *E/Z* isomers in the DMSO-*d*_6_ solution. The intensity ratio of the chemical resonances is 1:4 (**8a**) and 1.5:8.5 (**8b** and **8c**), and *Z* isomer is the prevailing one.

**Scheme 2:**
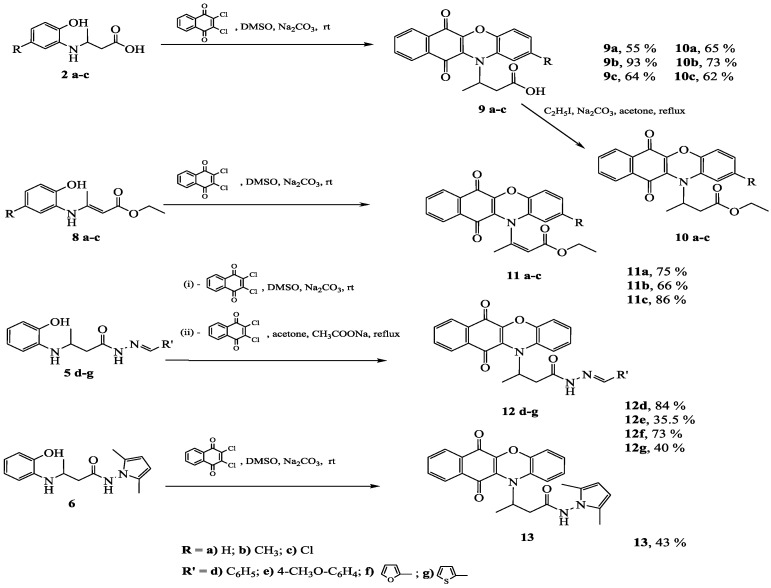
Synthesis of benzo[*b*]phenoxazine derivatives.

Condensation of compounds **2a–c**, **5d–g**, **6**, and **8a–c** with 2,3-dichloro-1,4-naphthoquinone by three different methods was investigated (Scheme 2).

3-(2-Substituted-6,11-dioxo-6,11-dihydro-12*H*-benzo[*b*]phenoxazin-12-yl)butanoic acids **9a–c** were prepared by the reactions of 3-[(2-hydroxyphenyl)amino]-butanoic acids **2a–c** with 2,3-dichloro-1,4-naphthoquinone in dimethylsulfoxide at room temperature in the presence of sodium carbonate. Reaction mixtures were diluted with water, filtered off and the filtrates were acidified with acetic acid up to pH 6. Acids **9a–c** are unstable under strong alkaline conditions and decompose to colourless compounds. Therefore, sodium carbonate was used in the work-up instead of sodium hydroxide. The synthesized compounds were purified by dissolving them in aqueous sodium carbonate solution and acidifying the filtrate with acetic acid to pH 6. The synthesis of 3-(6,11-dioxo-6,11-dihydro-12*H*-benzo[*b*]phenoxazin-12-yl)butanoic acid (**9a**) has been reported previously [34]. Its solution was found to stimulate the growth of barley at a concentration of 1.5 mg/L and it also increased the content of flavonoids.

Since compounds **9a–c** are unstable in acidic and alkaline media, they were treated with iodoetane in the presence of sodium carbonate to furnish ethyl 3-(2-substituted-6,11-dioxo-6,11-dihydro-12*H*-benzo[*b*]phenoxazin-12yl)butanoates **10a–c**.

The reactions of aminocrotonates **8a–c** and 2,3-dichloro-1,4-naphthoquinone in the presence of sodium carbonate afforded corresponding derivatives **11a–c**. In analogous way **12d** and **13** were synthesized from **5d** and **6**, respectively.

Some of the hydrazones did not react with 2,3-dichloro-1,4-naphthoquinone in DMSO. The attempts to synthesize the target products by the reaction in acetone, under reflux conditions, in the presence of sodium carbonate were unsuccessful. Therefore, sodium acetate was used as a base instead of sodium carbonate. Thus, *N*′-substituted-3-(6,11-dioxo-6,11-dihydro-12*H*-benzo[*b*]phenoxazin-12-yl)butanehydrazides **12e–g** were synthesized. Compounds **9a–c** could also be prepared by this method, but the reaction in DMSO was more facile, took place at room temperature and the work-up procedure was more simple. The ^1^H-NMR spectra have shown that in the DMSO-*d*_6_ solution compounds **11c** and **12d–g** exist as a mixture of *E/Z* isomers in the ratio 1:4 (**11c**) and 3:7 (**12d–g**) as reflected by the intensity of the resonance lines, and *Z* isomer is the prevailing one.

The structures of compounds **9–13** have been confirmed by the absence of the OH group proton singlets, which are present in the ^1^H-NMR spectra of the precursors **2a–c**, **5d–g**, **6**, and **8a–c**, and the increased intensity of the signals of the aromatic protons.

### 2.2. Biological Study

The synthesized compounds **5d–g**, **6**, **7a–c**, **9a–c**, **10a–c**, **11a–c**, **12d–g**, and **13** were evaluated for their antibacterial and antifungal activity against *Escherichia coli В-906*, *Staphylococcus aureus 209-Р*, *Mycobacterium luteum В-917*, *Candida tenuis VKM Y-70* and *Aspergillus niger VKM F-1119* strains by the diffusion [35] and serial dilution method (determination of minimum inhibitory concentration MIC) [36]. Their activity was compared to that of the known antibacterial agent vancomycin and the antifungal agent nystatin.

The test-culture *E. coli* had low sensitivity only to the compounds **9c** and **11b** (d = 8 mm) at a concentration of 0.5% (diffusion method). The compounds **9a–c**, **10c**, and **12f** had good activity against strain *S. aureus* at a concentration of 0.5% (diffusion method) and compounds **12d**, **12e**, **12g** were found to exhibit low antibacterial activity against *S. aureus*. The strain *M. luteum* was most sensitive to compounds **9a–c**, **10b,c**, **12f,g**, and **13** at a concentration of 0.5% and low sensitive to compounds **7b,c**. Other compounds had no antibacterial activity against *S. aureus* and *M. luteum* at 0.5% and 0.1% concentrations evaluated by the diffusion method. The results obtained are presented in Table 1.

**Table 1:** Antimicrobial activity of the synthesized compounds determined by diffusion method (only compounds that gave positive results at least in one case are included in the table).

Compound	Conc. (%)	Inhibition Diameter of Microorganism Growth (mm)				
		Bacteria			Fungi	
		*E. coli*	*S. aureus*	*M. luteum*	*C. tenuis*	*A. niger*
**7b**	0.5	0	0	13.0 (b/s)	0	12.0 (f/s)
	0.1	0	0	0	0	0
**7c**	0.5	0	0	16.0 (b/s)	0	10.0 (f/s)
	0.1	0	0	0	0	0
**9a**	0.5	0	22.4	15.0	12.0 (f/s)	0
	0.1	0	15.4	0	0	0
**9b**	0.5	0	24.4	19.4	0	0
	0.1	0	14.4	10.0 (b/s)	0	0
**9c**	0.5	8.0	23.4	23.0	20.0 (f/s)	0
	0.1	0	14.0	0	15.0 (f/s)	0
**10a**	0.5	0	0	7.0 (b/s)	0	0
	0.1	0	0	0	0	0
**10b**	0.5	0	0	11.4	0	0
	0.1	0	0	6.0	0	0
**10c**	0.5	0	23.7	20.7	0	0
	0.1	0	18.7	10.0 (b/s)	0	0
**11a**	0.5	0	0	0	10.0 (f/s)	0
	0.1	0	0	0	0	0
**11b**	0.5	8.0 (b/s)	0	0	0	0
	0.1	0	0	0	0	0
**12d**	0.5	0	8.0	0	0	0
	0.1	0	7.0 (b/s)	0	0	0
**12e**	0.5	0	7.7	0	0	0
	0.1	0	0	0	0	0
**12f**	0.5	0	24.0	15.4	0	0
	0.1	0	13.4	6.0	0	0
**12g**	0.5	0	11.7	13.0	0	24.4
	0.1	0	7.7	10.0	0	20.0
**13**	0.5	0	0	11.0	0	0
	0.1	0	0	0	0	0
C *	0.1	14.0	15.0	18.0	19.0	20.0

Notes: * Vancomycin was used as a control in the tests of antibacterial acitvity of the synthesized compounds, and nystatin was used in the tests of antifungal activity; b/s—bacteriostatic activity; f/s—fungistatic activity.

The results for the determination of minimum inhibitory concentrations (MIC) by the serial dilution method are provided in Table 2. All compounds, except **9c** and **11c** (500 µg/mL), showed no inhibition action against *E. coli*. Compounds **5d–g**, **6** didn’t show inhibition action against the *S. aureu*s bacterial strain at any concentration. Compounds **9b**, **9c**, **10c**, **12f** showed MIC against *S. aureus* at a concentration of 31.2 µg/mL. Compounds **9a**, **7b**, and **7a,c** were active at 62.5 µg/mL, 250 µg/mL, and 500 µg/mL, respectively. Compounds **9a–c**, **10b**, **12f** showed MIC against *M. luteum* at a concentration of 62.5 µg/mL, whereas MIC concentration for **13** was 15.6 µg/mL, the one for **10c** and **12g** was 31.2 µg/mL. Compounds **7a**, **c** were active at 250 µg/mL, and **5d,f**, **6**, **7b** showed MIC at 500 µg/mL.

**Table 2:** Bactericidal activity of the synthesized compounds determined by serial dilution method (only compounds that gave positive results at least in one case are included in the table).

Comp.	*E. coli*	*S. aureus*	*M. luteum*
	MIC (µg/mL)		
**5d**	+	+	500.0
**5f**	+	+	500.0
**6**	+	+	500.0
**7a**	+	500.0	250.0
**7b**	+	250.0	500.0
**7c**	+	500.0	250.0
**9a**	+	62.5	62.5
**9b**	+	31.2	62.5
**9c**	500.0	31.2	62.5
**10b**	+	+	62.5
**10c**	+	31.2	31.2
**11c**	500.0	+	+
**12f**	+	31.2	62.5
**12g**	+	+	31.2
**13**	+	+	15.6
C *	31.2	3.9	7.8

Notes: +: Growth of microorganisms; * Vancomycin was used as a control.

Antifungal activity (Table 1) (diffusion method) against *C. tenuis* was observed only for compounds **9a**, **9c**, and **11a** at a 0.5% concentration. The test-culture *A. niger* appeared to be insensitive to all compounds except 12 g at 0.5% and 0.1% concentrations. MIC against test-culture *C. tenuis* of **9b** and **9c** was observed at 31.2 µg/mL, the ones for **9a**, **6**, **11a**, and **11b** were observed at 15.6 µg/mL, 125 µg/mL, 250 µg/mL, and 500 µg/mL, respectively. Evaluation of antifungal activity of compounds showed that 12 g had MIC at 3.9 µg/mL, **11a**, **12f** at 62.5 µg/mL, and **7a**, **9c**, **11c**, **12e** at 125 µg/mL, **9a**, **10a** at 250 µg/mL for *A. niger*. For other compounds growth of fungi was observed at the investigated concentrations. Results are presented in Table 3.

**Table 3:** Fungicidal activity of the synthesized compounds determined by serial dilution method (only compounds that gave positive results at least in one case are included in the table).

Compound	*C. tenuis*	*A. niger*
	MIC (µg/mL^)^	
**6**	125.0	+
**7a**	+	125.0
**9a**	15.6	250.0
**9b**	31.2	+
**9c**	31.2	125.0
**10a**	+	250.0
**11a**	250.0	62.5
**11b**	500.0	+
**11c**	+	125.0
**12e**	+	125.0
**12f**	+	62.5
**12g**	+	3.9
C *	7.8	15.6

Notes: +: Growth of microorganisms; * Nystatin was used as a control.

The structure activity relationship study of these compounds has shown that introduction of the substituent into the benzo[*b*]phenoxazine moiety increases the antibacterial activity. 3-[(2-Hydroxyphenyl)amino]butanoic acid derivatives do not possess antimicrobial activity, whereas products of their condensation with 2,3-dichloro-1,4-naphthoquinone do exhibit antimicrobial action. The comparison of the activity of acids **9a–c** with the one of esters **10a–c** has shown that acids are more active against *S. aureus* and *M. luteum*. However, the presence of chlorine atom at the second position intensifies the antibacterial effect for esters, but does not influence the one for acids. On the other hand, esters **11a–c**, containing a double bond, are inactive against the tested bacteria strains and show moderate activity just against *A. niger*. As the comparison of benzo[*b*]phenoxazine derivatives with hydrazine moiety has revealed, the ones containing aromatic substituents are inactive against both bacteria and fungi. As it could be expected, benzo[*b*]phenoxazine derivatives containing hydrazide moiety with furan and thiophen substituents have shown significant antifungal activity against *A. niger*. Whereas, the pyrrole moiety has increased the activity against *M. luteum*.

## 3. Experimental Section

### 3.1. General Information

Melting points were determined on a Mel-Temp melting point apparatus (Electrochemical, A Bibby Scientific Company, Burlington, NJ, USA) and are uncorrected. The ^1^H- and ^13^C-NMR spectra were recorded on a Varian Unity Inova (300 MHz, 75 MHz) and AvanceIII 400 (400 MHz, 100 MHz) spectrometers operating in the Fourier transform mode, using TMS as an internal standard. Chemical shifts are expressed in parts per million (ppm, δ units). IR spectra (*ν*, cm^–1^) were recorded on a Perkin Elmer Spectrum BX FT-IR spectrometer using KBr tablets. Mass spectra were obtained on a Waters ZQ 2000 spectrometer (Micromass, Milford, MA, USA) using ESI technique. TLC was performed on Fluka Silica gel plates on TLC Alu foils, 60 Å, F_254_. Silica gel (Fluka, 230–400 mesh particle size, pore size 60 Å) was used for column chromatography.

### 3.2. Chemistry

*3-[(2-Hydroxy-5-substitutedphenyl)amino]butanoic acids* (**2a–c**) were prepared as described in [37]. Their melting points and ^1^H-NMR spectra were identical to those described in [37].

*Methyl 3-[(2-hydroxyphenyl)amino]butanoate* (**3**): A mixture of 3-[(2-hydroxyphenyl)amino]butanoic acid **2a** (1.95 g, 0.01 mol), methanol (20 mL) and catalytic amount of conc. H_2_SO_4_ was refluxed for 6 h. Then the solvent was removed under reduced pressure. The precipitate was neutralized with 10% Na_2_CO_3_ solution. The crude product was extracted with diethyl ether. Yield 0.636 g (33%); liquid; R_f_ = 0.65 (acetone–hexane, 1:1); IR (KBr), *ν*, cm^−1^: 3391 (OH), 2955 (NH), 2730 (OCH_3_), 1732, 1715 (CO); ^1^H-NMR (400 MHz, DMSO-*d*_6_): δ = 1.17 (d, 3H, *J* = 6.4 Hz, CH_3_), 2.44 (dd, 1H, *J* = 6.9, 15.0 Hz, CH_2_), 2.63 (dd, 1H, *J* = 6.9, 15.0 Hz, CH_2_), 3.60 (s, 3H, OCH_3_), 3.87–3.77 (m, 1H, *CH*CH_2_), 4.45 (d, 1H, *J* = 9.7 Hz, NH), 6.41–6.68 (m, 4H, H_aromatic_), 9.26 (s, 1H, OH). Anal. Calcd. (%) for C_11_H_15_NO_3_: C, 63.14; H, 7.23; N, 6.69%. Found: C, 63.24; H, 7.31; N, 6.52%.

*3-[(2-hydroxyphenyl)amino]butanehydrazide* (**4**). *Method A*: A mixture of acid **2a** (1.95 g, 0.01 mol) and 98% hydrazide hydrate (1.25 g, 0.025 mol) in toluene (30 mL) was refluxed for 14 h. The residual semisolid obtained was used in subsequent reactions without further purification. *Method B*: A mixture of ester **3** (0.636 g, 3.3 mmol), 2-propanol (20 mL) and 98% hydrazine hydrate (0.25 g, 5 mmol) was refluxed for 5 h. The reaction mixture was cooled, the liquid fraction was decanted and oily residue was obtained.

#### 3.2.1. General Procedure for Synthesis of *N'*-Aryl-3-[(2-hydroxyphenyl)amino]butanehydrazides (**5d–g**)

A mixture of 3-[(2-hydroxyphenyl)amino]butanehydrazide (**4**), corresponding arylaldehyde (0.015 mol) and 2-propanol (30 mL) was heated under reflux for 3 h. The reaction mixture was cooled down, the precipitate was filtered off and washed with 2-propanol and diethyl ether or the solvent was evaporated under reduced pressure and the product was purified by column chromatography (acetone–hexane, 1:1).

*N'-Benzylidene-3-[(2-hydroxyphenyl)amino]butanehydrazide* (**5d**). Yield 0.25 g (13.5%), m.p.: 92–93 °C; R_f_ = 0.58 (acetone–hexane, 1:1); IR (KBr), *ν*, cm^−1^: 3396 (OH), 2976 (CONH), 2927 (NH), 1648 (CO); ^1^H-NMR (400 MHz, DMSO-*d*_6_): δ = 1.18 (d, (1.2)3H (*E*), *J* = 6.3 Hz, CH_3_), 1.22 (d, (1.8)3H (*Z*), *J* = 6.3 Hz, CH_3_), 2.64 (dd, 1H, *J* = 7.6, 14.4 Hz, CH_2_), 3.08 (dd, 1H, *J* = 5.4, 14.3 Hz, CH_2_), 3.84–3.94 (m, 1H, *CH*CH_2_), 4.53 (d, 1H, *J* = 11.9 Hz, NH), 6.40–6.67 (m, 4H, H_aromatic_), 7.42–7.69 (m, 5H, H_aromatic_), 8.01 (s, (0.6)1H, (*Z*), NCH), 8.18 (s, (0.4)1H (*E)*, NCH), 9.22 (s, (0.6)1H (*Z*), OH) 9.25 (s, (0.4)1H (*E*), OH), 11.35 (s, (0.6)1H (*Z*), NHN), 11.43 (s, (0.4)1H (*E*), NHN); ^13^C-NMR (100 MHz, DMSO-*d*_6_): δ = 20.31 (CH_3_ (*E*)), 20.45 (CH_3_ (*Z*)), 41.22 (CH_2_), 45.41 (CH), 110.49, 110.66, 113.56, 115.88, 115.97, 119.70, 119.76, 126.78, 127.02, 128.84, 129.76, 129.97, 134.29, 136.08, 144.27 (C_aromatic_), 142.98 (NCH (*Z*)), 146.13 (NCH (*E*)), 167.18 (CO (*E*)), 172.92 (CO (*Z*)); HRMS calculated for C_17_H_19_N_3_O_2_ [M+H]^+^ 298.1477 found 298.1551; Anal. Calcd. (%) for: C, 68.67; H, 6.44; N, 14.13, found: C, 68.70; H, 6.88; N, 14.31.

*3-[(2-Hydroxyphenyl)amino]-N'-[4-methoxybenzylidene]butanehydrazide* (**5e**). Yield 0.47 g (23%); m.p.: 163–164 °C; IR (KBr), *ν*, cm^−1^: 3433 (OH), 2968 (CONH), 2968 (NH), 2838 (OCH_3_), 1619, 1602 (CO); ^1^H-NMR (400 MHz, DMSO-*d*_6_): δ = 1.15 (d, (1.2)3H (*E*), *J* = 6.4 Hz, CH_3_), 1.21 (d, (1.8)3H (*Z*), *J* = 6.6 Hz, CH_3_), 2.60 (dd, 1H, *J* = 7.5, 14.7 Hz, CH_2_), 3.05 (dd, 1H, *J* = 5.4, 14.3 Hz, CH_2_), 3.82 (s, 3H, OCH_3_), 3.90–3.95 (m, 1H, *CH*CH_2_), 4.52 (d, 1H, *J* = 11.9 Hz, NH), 6.39–6.68 (m, 4H, H_aromatic_), 7.58–7.63 (m, 4H, H_aromatic_), 8.32 (s, (0.6)1H, (*Z*), NCH), 8.43 (s, (0.4)1H (*E)*, NCH), 9.21 (s, (0.6)1H (*Z*), OH) 9.25 (s, (0.4)1H (*E*), OH), 11.22 (s, (0.6)1H (*Z*), NHN), 11.29 (s, (0.4)1H (*E*), NHN); HRMS calculated for C_18_H_21_N_3_O_3_ [M+H]^+^ 328.1583 found 328.1585; Anal. Calcd. (%) for: C, 66.04; H, 6.47; N, 12.84, found: C, 66.10; H, 6.38; N, 12.91.

*N'-[Furan-2-ylmethylene]-3-[(2-hydroxyphenyl)amino]butanehydrazide* (**5f**). Yield 0.334 g (19%); m.p.: 175–176 °C; IR (KBr), *ν*, cm^−1^: 3401 (OH), 2974 (CONH), 2934 (NH), 1628, 1609 (CO); ^1^H-NMR (400 MHz, DMSO-*d*_6_): δ = 1.14 (d, (1.2)3H (*E*), *J* = 6.3 Hz, CH_3_), 1.16 (d, (1.8)3H (*Z*), *J* = 6.3 Hz, CH_3_), 2.73 (dd, 1H, *J* = 7.5, 14.7 Hz, CH_2_), 3.02 (dd, 1H, *J* = 5.1, 14.9 Hz, CH_2_), 3.76–3.87 (m, 1H, *CH*CH_2_), 4.60 (d, 1H, *J* = 11.0 Hz, NH), 5.84 (s, 1H, CH), 6.39–6.67 (m, 4H, H_aromatic_), 6.87 (s, 1H, CH), 7.82 (s, (0.6)1H (*Z*), NCH), 7.89 (s, (0.4)1H (*E*), NCH), 8.07 (s, 1H, CH), 9.30 (br. s, 1H, OH), 11.29 (s, (0.6)1H (*Z*), NHN), 11.40 (s, (0.4)1H (*E*), NHN); HRMS calculated for C_15_H_17_N_3_O_3_ [M+H]^+^ 288.1270 found 288.1345; Anal. Calcd. (%) for: C, 62.71; H, 5.96; N, 14.63, found: C, 62.60; H, 5.88; N, 14.71.

*3-[(2-Hydroxyphenyl)amino]-N'-[thiophen-2-ylmethylene]butanehydrazide* (**5g**). Yield 0.44 g (23%); m.p.: 95–96 °C; IR (KBr), *ν*, cm^−1^: 3199 (OH), 3072 (CONH), 2970 (NH), 1660 (CO); ^1^H-NMR (400 MHz, DMSO-*d*_6_): δ = 1.17 (d, (1.2)3H (*E*), *J* = 6.3 Hz, CH_3_), 1.20 (d, (1.8)3H (*Z*), *J* = 6.4 Hz, CH_3_), 2.28 (m, 1H, CH_2_), 3.01 (dd, 1H, *J* = 5.4, 14.2 Hz, CH_2_), 3.73–3.88 (m, 1H, *CH*CH_2_), 4.49 (d, 1H, *J* = 11.8 Hz, NH), 6.2–6.67 (m, 4H, H_aromatic_), 7.11 (s, 1H, CH), 7.63 (s, 1H, CH), 8.18 (s, (0.6)1H (*Z*), NCH) , 8.19 (s, (0.4)1H (*E*), NCH), 8.4 (s, 1H, CH), 9.22 (br. s, 1H, OH), 11.30 (s, (0.6)1H (*Z*), NHN), 11.38 (s, (0.4)1H (*E*), NHN); HRMS calculated for C_15_H_17_N_3_O_2_S [M+H]^+^ 304.1041 found 304.2612; Anal. Calcd. (%) for: C, 59.39; H, 5.65; N, 13.85, found: C, 59.70; H, 5.86; N, 13.61.

*N-(2,5-Dimethyl-1H-pyrrol-1-yl)-3-[(2-hydroxyphenyl)amino]butanamide* (**6**): A mixture of 3-[(2-hydroxyphenyl)amino]butanehydrazide (**4**), hexane-2,5-dione (2.28 g, 0.02 mol) and 2-propanol (20 mL) was heated under reflux for 6 h. The solvent was evaporated under reduced pressure and the product was purified by column chromatography (acetone–hexane, 1:1). Yield 0.35 g (19.5%); m.p.: 154–155 °C; R_f_ = 0.28 (Ac:H, 1:2); IR (KBr), *ν*, cm^−1^: 3296 (OH), 2974 (CONH), 2866 (NH), 1686, 1654 (CO); ^1^H-NMR (400 MHz, DMSO-*d*_6_): δ = 1.22 (d, 3H, *J* = 6.3 Hz, CH_3_), 1.94 (d, 6H, *J* = 20.1 Hz, 2CH_3_), 2.39 (dd, 1H, *J* = 6.9, 14.0 Hz, CH_2_), 2.59 (dd, 1H, *J* = 5.9, 14.0 Hz, CH_2_), 3.90 (m, 1H, *CH*CH_2_), 4.53 (d, 1H, *J* = 11.9 Hz, NH), 5.62 (s, 2H, 2CH), 6.41–6.68 (m, 4H, H_aromatic_), 9.26 (s, 1H, OH), 10.68 (s, 1H, NHN); ^13^C-NMR (100 MHz, DMSO-*d*_6_): δ = 11.45 (2CH_3_), 20.65 (CH_3_), 40.58 (CH_2_), 45.79 (CH), 103.38 (2CH), 110.93, 114.06, 116.45, 120.16, 136.35, 144.81 (C_aromatic_), 127.10, 127.31 (NC), 170.58 (CO); HRMS calculated for C_16_H_21_N_3_O_2_ [M+H]^+^ 288.1634 found 288.1707; Anal. Calcd. (%) for: C, 66.88; H, 7.37; N, 14.62, found: C, 66.70; H, 7.28; N, 14.51.

#### 3.2.2. General Procedure for Synthesis of 3-[(2-[{3-Chloroquinoxalin-2-yl}oxy]-5-substitutedphenyl)amino]butanoic Acids **7a–c**

A mixture of corresponding acid **2a–c** (8 mmol), 2,3-dichloroquinoxaline (2.98 g, 15 mmol), KOH (0.84 g, 15 mmol), and acetone (40 mL) was heated under reflux for 20 h, then cooled down and diluted with water (80 mL). Undissolved precipitate was filtered off and the solution was acidified with acetic acid to pH 6. The crystals were filtered off, washed with water, and recrystallized from 2-propanol.

*3-[(2-((3-Chloroquinoxalin-2-yl)oxy)phenyl)amino]butanoic acid* (**7a**). Yield 2.31 g (84%); m.p.: 201–202 °C; IR (KBr), *ν*, cm^−1^: 3105 (OH), 2974 (NH), 1731 (CO); ^1^H-NMR (300 MHz, DMSO-*d*_6_): δ = 1.60 (d, 3H, *J* = 6.9 Hz, CH_3_), 2.98–3.06 (dd, 1H, *J* = 7.3, 16.3 Hz, CH_2_), 3.16–3.24 (dd, 1H, *J* = 6.5, 16.3 Hz, CH_2_), 5.04–5.12 (m, 1H, CH), 6.92–7.58 (m, 8H, H_aromatic_), 12.32 (br s, 1H, OH); ^13^C-NMR (75 MHz, DMSO-*d*_6_): δ = 16.67 (CH_3_), 37.29 (CH_2_), 48.39 (CH), 99.42, 114.60, 116.31, 122.60, 124.87, 125.75, 126.08, 126.18, 127.71, 136.57, 139.22, 141.05, 142.61, 147.78 (C_aromatic_), 172.58 (CO). Anal. Calcd. (%) for C_18_H_16_ClN_3_O_3_: C, 60.42; H, 4.51; N, 11.74, found: C, 60.84; H, 4.74; N, 12.05.

*3-[(2-((3-Chloroquinoxalin-2-yl)oxy)-5-methylphenyl)amino]butanoic acid* (**7b**). Yield 1.17 g (67%); m.p.: 217–219 °C; IR (KBr), *ν*, cm^−1^: 2973 (OH); 2922 (NH); 1708 (CO); ^1^H-NMR (300 MHz, DMSO-*d*_6_): δ = 1.60 (d, 3H, *J* = 6.9 Hz, CH*CH_3_*), 2.27 (s, 3H, CH_3_), 3.01–3.09 (dd, 1H, *J* = 7.2, 16.3 Hz, CH_2_), 3.22–3.30 (dd, 1H, *J* = 6.8, 16.3 Hz, CH_2_), 5.00–5.07 (m, 1H, CH), 6.73 (d, 1H, *J* = 8.9 Hz, NH), 6.86–7.57 (m, 7H, H_aromatic_), 12.28 (s, 1H, OH); ^13^C-NMR (75 MHz, DMSO-*d*_6_): δ = 16.70 (CH*CH_3_*), 20.61 (CH_3_), 37.15 (CH_2_), 48.47 (CH), 114.87, 116.01, 122.84, 125.73, 126.05, 126.14, 127.62, 129.50, 134.13, 136.57, 139.14, 140.44, 140.99, 147.85 (C_aromatic_), 172.48 (CO). Anal. Calcd. (%) for C_19_H_18_ClN_3_O_3_: C, 61.38; H, 4.88; N, 11.30, found: C, 61.07; H, 5.10; N, 11.42.

*3-[(5-Chloro-2-[(3-chloroquinoxalin-2-yl)oxy]phenyl)amino]butanoic acid* (**7c**). Yield 1.58 g (77%); m.p.: 195–196 °C; IR (KBr), ν, cm^−1^: 3066 (OH), 2975 (NH), 1707 (CO); ^1^H-NMR (300 MHz, DMSO-*d*_6_): δ = 1.58 (d, 3H, *J* = 6.9 Hz, CH_3_), 2.94–3.02 (dd, 1H, *J* = 6.7, 16.4 Hz, CH_2_), 3.28–3.36 (dd, 1H, *J* = 7.1, 16.4 Hz, CH_2_), 4.91–4.98 (m, 1H, CH), 6.93 (d, 1H, *J* = 8.5 Hz, NH), 7.00–7.60 (m, 7H, H_aromatic_), 12.33 (s, 1H, OH); ^13^C-NMR (75 MHz, DMSO-*d*_6_): δ = 16.72 (CH_3_), 37.27 (CH_2_), 49.21 (CH), 114.53, 117.54, 121.91, 125.95, 126.27, 126.47, 127.90, 128.66, 136.59, 139.04, 140.49, 141.63, 147.49, 151.20 (C_aromatic_), 172.62 (CO). Anal. Calcd. (%) for C_18_H_15_Cl_2_N_3_O_3_: C, 55.12; H, 3.85; N, 10.71, found: C, 55.84; H, 4.10; N, 10.78.

#### 3.2.3. General Procedure for Synthesis of Ethyl-3-[(2-Hydroxy-5-substitutedphenyl)amino]but-2-enoates (**8a–c**)

A mixture of corresponding 2-aminophenol **1a–c** (0.1 mol), ethyl 3-oxobutanoate (19.5 g, 0.15 mol), acetic acid (0.5 mL) and toluene (100 mL) was heated under reflux for 3 h. Water generated during the course of the reaction was removed with Dean-Stark trap, the liquid fraction was evaporated under reduced pressure. Residue was poured over with hexane (150 mL) and heated at reflux temperature. The precipitate was filtered off and recrystallized from 2-propanol.

*Ethyl-3-[(2-hydroxyphenyl)amino]but-2-enoate* (**8a**). Yield 10.15 g (47%); m.p.: 95–96 °C IR (KBr), *ν*, cm^−1^: 3232 (OH), 2978 (NH), 1636 (CO); ^1^H-NMR (300 MHz, DMSO-*d*_6_): δ = 1.08 (t, (0.2)3H (*E*), *J* = 7.1 Hz, OCH_2_*CH_3_*), 1.18 (t, (0.8)3H (*Z*), *J* = 7.1 Hz, OCH_2_*CH_3_*), 1.98 (s, (0.8)3H (*Z*), =CCH_3_), 2.32 (s, (0.2)3H (*E*), =CCH_3_), 3.88 (q, (0.2)2H (*E*), *J* = 7.1 Hz, O*CH_2_*CH_3_), 4.04 (q, (0.8)3H (*Z*), *J* = 7.1 Hz, O*CH_2_*CH_3_), 4.44 (s, (0.2)1H (*E*), =CH), 4.64 (s, (0.8)1H (*Z*), =CH), 6.74–7.16 (m, 4H, H_aromatic_), 7.94 (s, (0.2)1H (*E*), OH), 9.64 (s, (0.2)1H (*E*), NH), 9.86 (s, (0.8)1H (*Z*), NH), 10.18 (s, (0.8)1H (*Z*), OH). Anal. Calcd. (%) for C_12_H_15_NO_3_: C, 65.14; H, 6.83; N, 6.33, found: C, 65.53; H, 7.03; N, 6.44.

*Ethyl-3-[(2-hydroxy5-methylphenyl)amino]but-2-enoate* (**8b**). Yield 11.90 g (51%); m.p.: 103–104 °C; IR (KBr), *ν*, cm^−1^: 3286 (OH), 3142 (NH), 1622 (CO); ^1^H-NMR (300 MHz, DMSO-*d*_6_): δ = 1.08 (t, (0.15)3H (*E*), *J* = 7.1 Hz, OCH_2_*CH_3_*), 1.18 (t, (0.85)3H (*Z*), *J* = 7.1 Hz, OCH_2_*CH_3_*), 1.97 (s, (0.85)3H (*Z*), =CCH_3_), 2.00 (s, (0.15)3H (*E*), =CCH_3_), 2.19 (s, (0.85)3H (*Z*), 4-CH_3_), 2.30 (s, (0.15)3H (*E*), 4-CH_3_), 3.88 (q, (0.15)2H (*E*), *J* = 7.1 Hz, O*CH_2_*CH_3_), 4.03 (q, (0.85)3H (*Z*), *J* = 7.1 Hz, O*CH_2_*CH_3_), 4.41 (s, (0.15)1H (*E*), =CH), 4.62 (s, (0.85)1H (*Z*), =CH), 6.71–6.96 (m, 3H, H_aromatic_), 7.36, 7.90, 8.88, 9.19, 9.53, 9.59, 9.81, 10.13, 10.64 (9s, 2H, OH+NH). Anal. Calcd. (%) for C_13_H_17_NO_3_: C, 66.36; H, 7.28; N, 5.95, found: C, 66.07; H, 7.30; N, 6.37.

*Ethyl-3-[(5-chloro-2-hydroxyphenyl)amino]but-2-enoate* (**8c**). Yield 13.3 g (52%); m.p.: 134–136 °C; IR (KBr), *ν*, cm^−1^: 3250 (OH), 3159 (NH), 1620 (CO); ^1^H-NMR (300MHz, DMSO-*d*_6_): δ = 1.09 (t, (0.15)3H (*E*), *J* = 7.1 Hz, OCH_2_*CH_3_*), 1.18 (t, (0.85)3H (*Z*), *J* = 7.1 Hz, OCH_2_*CH_3_*), 2.05 (d, (0.85)3H (*Z*), =CCH_3_), 2.07 (d, (0.15)3H (*E*), *J* = 0.6 Hz, =CCH_3_), 3.90 (q, (0.15)3H (*E*), *J* = 7.1 Hz, O*CH_2_*CH_3_), 4.04 (q, (0.85)3H (*Z*), *J* = 7.1 Hz, O*CH_2_*CH_3_), 4.47 (q, (0.15)1H (*E*), *J* = 0.6 Hz, =CH), 4.84 (q, (0.85)1H (*Z*), *J* = 0.6 Hz, =CH), 6.81–7.00 (m, 3H, H_Ar_), 8.78, 9.24, 9.55, 9.82, 10.10, 10.20, 10.23, 10.27, 10.92 (9s, 2H, OH+NH). Anal. Calcd. (%) for C_12_H_14_ClNO_3_: C, 56.37; H, 5.52; N, 5.48, found: C, 56.00; H, 5.80; N, 5.73.

#### 3.2.4. General Procedure for Synthesis of 3-(2-Substituted-6,11-dioxo-6,11-dihydro-12*H*-benzo[*b*]phenoxazin-12-yl)butanoic Acids **9a–c**

A mixture of the corresponding compound **2a–c** (0.05 mol), 2,3-dichloro-1,4-naphthoquinone (11.35 g, 0.05 mol), sodium carbonate (2.01 g, 0.019 mol), and dimethyl sulfoxide (20 mL) was stirred at room temperature for 14 h. The reaction was quenched by diluting the reaction mixture with water, causing the product to precipitate. The crude product was isolated by dissolving it in aqueous sodium carbonate solution and acidifying filtrate with acetic acid up to pH 6.

*3-(6,11-Dioxo-6,11-dihydro-12H-benzo[b]phenoxazin-12-yl)butanoic acid* (**9a**). Yield 0.95 g (55%); its melting point and ^1^H- and ^13^C-NMR spectra were identical to those described in [31].

*3-(2-Methyl-6,11-dioxo-6,11-dihydro-12H-benzo[b]phenoxazin-12-yl)butanoic acid* (**9b**). Yield 4.73 g (93%); m.p.: 169–170 °C; IR (KBr), *ν*, cm^−1^: 3312 (OH), 1724, 1673, 1625 (CO); ^1^H-NMR (300 MHz, DMSO-*d*_6_): δ = 1.49 (d, 3H, *J* = 6.9 Hz, CH*CH_3_*), 2.22 (s, 3H, CH_3_), 2.59–2.67 (dd, 1H, *J* = 7.5, 16.3 Hz, CH_2_), 2.81–2.89 (dd, 1H, *J* = 6.8, 16.3 Hz, CH_2_), 4.08–4.19 (m, 1H, CH), 6.75–7.95 (m, 7H, H_aromatic_), 12.25 (s, 1H, OH); ^13^C-NMR (75 MHz, DMSO-*d*_6_): δ = 20.38 (CH*CH_3_*), 20.67 (CH_3_), 56.91 (CH_2_), 115.82 (CH), 120.63, 125.09, 125.14, 125.93, 129.95, 130.72, 131.33, 133.65, 133.92, 134.33, 136.22, 136.34, 145.45, 145.83 (2C+C_aromatic_), 172.57, 175.81, 180.91 (3CO). Anal. Calcd. (%) for C_21_H_17_NO_5_: C, 69.41; H, 4.72; N, 3.85, found: C, 69.43; H, 4.80; N, 3.79.

*3-(2-Chloro-6,11-dioxo-6,11-dihydro-12H-benzo[b]phenoxazin-12-yl)butanoic acid* (**9c**). Yield 3.12 g (64%); m.p.: 214–215 °C; IR (KBr), *ν*, cm^−1^: 3195 (OH), 1723, 1669, 1642 (CO); ^1^H-NMR (300 MHz, DMSO-*d*_6_): δ = 1.46 (d, 3H, *J* = 6.9 Hz, CH_3_), 2.53–2.61 (dd, 1H, *J* = 7.7, 16.1 Hz, CH_2_), 2.75–2.83 (dd, 1H, *J* = 6.7, 16.1 Hz, CH_2_), 4.08–4.19 (m, 1H, CH), 6.87–7.93 (m, 7H, H_aromatic_), 12.48 (s, 1H, OH); ^13^C-NMR (75 MHz, DMSO-*d*_6_): δ = 20.69 (CH_3_), 57.59 (CH_2_), 61.70 (CH), 99.44, 117.36, 119.37, 124.38, 125.17, 126.01, 128.56, 129.85, 130.70, 133.71, 133.79, 134.02, 135.50, 145.32 (2C+C_aromatic_), 146.99, 175.69, 180.67 (3CO). Anal. Calcd. (%) for C_20_H_14_ClNO_5_: C, 62.59; H, 3.68; N, 3.65, found: C, 62.47; H, 3.82; N, 3.63.

#### 3.2.5. General Procedure for Synthesis of Ethyl 3-(2-substituted-6,11-dioxo-6,11-dihydro-12*H*-benzo[*b*]phenoxazin-12yl)butanoates **10a–c**

A mixture of corresponding butanoic acid **9a–c** (0.033 mol), iodoethane (1.6 mL, 0.02 mol), potassium carbonate (1.52 g, 0.011 mol), and acetone (10 mL) was heated under reflux for 3.5 h. The liquid fraction was removed under reduced pressure. Water (20 mL) was poured over the residue; the precipitate was filtered off and washed with water.

*Ethyl 3-(6,11-dioxo-6,11-dihydro-12H-benzo[b]phenoxazin-12-yl)butanoate* (**10a**). Yield 0.8 g (65%); m.p.: 163–165 °C; IR (KBr), *ν*, cm^−1^: 1726, 1657, 1630 (CO); ^1^H-NMR (300 MHz, CDCl_3_): δ = 1.19 (t, 3H, *J* = 7.1 Hz, CH_2_*CH_3_*), 1.57 (d, 3H, *J* = 6.9 Hz, CH_3_), 2.63–2.71 (dd, 1H, *J* = 7.4, 15.9 Hz, CH*CH_2_*), 3.00–3.06 (dd, 1H, *J* = 7.1, 15.9 Hz, CH*CH_2_*), 4.10–4.13 (m, 2H, CH_2_), 4.24–4.35 (m, 1H, CH), 6.92–8.07 (m, 8H, H_aromatic_); ^13^C-NMR (75 MHz, CDCl_3_): δ = 14.05 (CH_2_*CH_3_*), 21.24 (CH_3_), 40.43 (CH*CH_2_*), 57.47 (CH_2_), 60.80 (CH), 116.87, 120.16, 124.94, 125.28, 125.88, 126.40, 130.38, 131.04, 131.88, 133.47, 133.70, 136.53, 146.54, 149.02 (2C+C_aromatic_), 171.18, 176.65, 181.21 (3CO). Anal. Calcd. (%) for C_22_H_19_NO_5_: C, 70.02; H, 5.07; N, 3.71, found: C, 68.05; H, 4.85; N, 3.62.

*Ethyl 3-(2-methyl-6,11-dioxo-6,11-dihydro-12H-benzo[b]phenoxazin-12-yl)butanoate* (**10b**). Yield 0.77 g (73%); m.p.: 88–90 °C; IR (KBr), *ν*, cm^−1^: 1721, 1664, 1655 (CO); ^1^H-NMR (300 MHz, DMSO-*d*_6_): δ = 1.06 (t, 3H, *J* = 7.1 Hz, CH_2_*CH_3_*), 1.50 (d, 3H, *J* = 6.8 Hz, CH*CH_3_*), 2.22 (s, 3H, CH_3_), 2.69–2.77 (dd, 1H, *J* = 6.8, 16.0 Hz, CH*CH_2_*), 2.85–2.93 (dd, 1H, *J* = 7.5, 16.0 Hz, CH*CH_2_*), 3.96–4.01 (m, 2H, CH_2_), 4.03–4.12 (m, 1H, CH), 6.74–7.93 (m, 7H, H_aromatic_); ^13^C-NMR (75 MHz, DMSO-*d*_6_): δ = 13.76 (CH_2_*CH_3_*), 20.36 (CH*CH_3_*), 20.98 (CH_3_), 30.59 (CH_2_), 60.04 (CH), 83.83, 98.55, 98.94, 99.41, 103.27, 115.81, 120.87, 120.71, 125.27, 131.11, 134.42, 140.96, 146.26, 170.74 (2C+C_aromatic_), 171.09, 179.88, 198.75 (3CO). Anal. Calcd. (%) for C_23_H_21_NO_5_: C, 70.58; H, 5.41; N, 3.58, found: C, 70.25; H, 5.53; N, 3.41.

*Ethyl 3-(2-chloro-6,11-dioxo-6,11-dihydro-12H-benzo[b]phenoxazin-12-yl)butanoate* (**10c**). Yield 0.66 g (62%); m.p.: 86–88 °C; IR (KBr), *ν*, cm^−1^: 1731, 1666, 1625 (CO); ^1^H-NMR (300 MHz, DMSO-*d*_6_): δ = 1.07 (t, 3H, *J* = 7.1 Hz, CH_2_*CH_3_*), 1.49 (d, 3H, *J* = 6.9 Hz, CH_3_), 2.72–2.80 (dd, 1H, *J* = 6.9, 16.2 Hz, CH*CH_2_*), 2.85–2.92 (dd, 1H, *J* = 7.7, 16.2 Hz, CH*CH_2_*), 3.98–4.02 (m, 2H, CH_2_), 4.12–4.19 (m, 1H, CH), 6.89–7.94 (m, 7H, H_aromatic_); ^13^C-NMR (75 MHz, DMSO-*d*_6_): δ = 13.75 (CH_2_*CH_3_*), 20.68 (CH_3_), 30.08 (CH*CH_2_*), 57.27 (CH_2_), 60.11 (CH), 117.40, 119.57, 124.62, 125.16, 125.19, 126.14, 128.69, 133.31, 133.86, 134.06, 135.18, 144.27, 145.57, 147.03 (2C+C_aromatic_), 170.64, 175.75, 180.59 (3CO). Anal. Calcd. (%) for C_22_H_18_ClNO_5_: C, 64.16; H, 4.41; N, 3.40, found: C, 64.59; H, 3.96; N, 3.51.

#### 3.2.6. General Procedure for the Synthesis of **11a–c**, **12d** and **13**

A mixture of **8a–c**, **5d** or **6** (0.33 mmol), 2,3-dichloro-1,4-naphthoquinone (0.075 g, 0.33 mmol), sodium carbonate (0.20 g, 1.9 mmol), and DMSO (20 mL) was stirred at room temperature for 17 h. The reaction was quenched by diluting the reaction mixture with water, causing the products to precipitate. The precipitate was filtered off and washed with water. Compounds **11a–c** were recrystallized from acetone. Compounds **12d** and **13** were purified by column chromatography (acetone–hexane, 1:1).

*Ethyl-3-(6,11-dioxo-6,11-dihydro-12H-benzo[b]phenoxazin-12-yl)but-2-enoate* (**11a**) was synthesized from **8a** to afford 0.70 g (75%) of **11a**; m.p.: 193–194 °C; IR (KBr), *ν*, cm^−1^: 1712, 1671, 1639 (CO); ^1^H-NMR (300 MHz, DMSO-*d*_6_): δ = 1.19 (t, 3H, *J* = 7.1 Hz, OCH_2_*CH_3_*), 2.42 (s, 3H, =CCH_3_), 4.10 (q, 2H, *J* = 7.1 Hz, O*CH_2_*CH_3_), 5.88 (s, 1H, =CH), 6.92–7.02 (m, 4H, H_aromatic_); 7.77–7.97 (m, 4H, H_aromatic_); ^13^C-NMR (75 MHz, DMSO-*d*_6_): δ = 14.12 (OCH_2_*CH_3_*), 18.69 (=C*CH_3_*), 59.62 (O*CH_2_*CH_3_), 112.72 (=CH), 116.83, 118.77, 125.40, 125.29, 125.88, 126.02, 130.04, 130.24, 130.59, 130.85, 131.08, 134.22, 144.48, 145.88 (2C+C_aromatic_), 159.83 (=*C*CH_3_), 165.89, 175.90, 178.81 (3CO). Anal. Calcd. (%) for C_22_H_17_NO_5_: C, 70.39; H, 4.56; N, 3.73, found: C, 70.13; H, 4.79; N, 3.70.

*Ethyl-3-(2-methyl-6,11-dioxo-6,11-dihydro-12H-benzo[b]phenoxazin-12-yl)but-2-enoate* (**11b**) was synthesized from **8b** to afford 2.32 g (66%) of **11b**; m.p.: 207–209 °C; IR (KBr), *ν*, cm^−1^: 1710, 1670, 1645 (CO); ^1^H-NMR (300 MHz, DMSO-*d*_6_): δ = 1.20 (t, 3H, *J* = 7.1 Hz, OCH_2_*CH_3_*), 2.19 (s, 3H, CH_3_), 2.43 (s, 3H, =CCH_3_), 4.10 (q, 2H, *J* = 7.1 Hz, O*CH_2_*CH_3_), 5.86 (s, 1H, =CH), 6.75–6.88, 7.80–7.97 (m, 7H, m, H_aromatic_). Anal. Calcd. (%) for C_23_H_19_NO_5_: C, 70.94; H, 4.92; N, 3.60, found: C, 71.03; H, 5.02; N, 3.75.

*Ethyl-3-(2-chloro-6,11-dioxo-6,11-dihydro-12H-benzo[b]phenoxazin-12-yl)but-2-enoate* (**11c**) was synthesized from **8c** to afford 3.08 g (86%) of **11c**; m.p.: 208–209 °C; IR (KBr), *ν*, cm^−1^: 1712, 1671, 1646 (CO); ^1^H-NMR (300 MHz, DMSO-*d*_6_): δ = 1.05 (t, (0.8)3H (*Z*), *J* = 7.1 Hz, OCH_2_*CH_3_*), 1.21 (t, (0.2)3H (*E*), *J* = 7.1 Hz, OCH_2_*CH_3_*), 2.22 (d, (0.8)3H (*Z*), *J* = 1.0 Hz, =CCH_3_), 2.41 (d, (0.2)3H (*E*), *J* = 1.0 Hz, =CCH_3_), 4.00 (q, (0.8)2H (*Z*), *J* = 7.1 Hz, O*CH_2_*CH_3_), 4.13 (q, (0.2)2H (*E*), *J* = 7.1 Hz, O*CH_2_*CH_3_), 6.01 (q, (0.2)1H (*E*), *J* = 1.0 Hz, =CH), 6.24 (q, (0.8)1H (*Z*), *J* = 1.0 Hz, =CH), 6.41–6.98, 7.72–7.94 (m, 7H, H_aromatic_). Anal. Calcd. (%) for C_22_H_16_ClNO_5_: C, 64.48; H, 3.94; N, 3.42, found: C, 64.97; H, 4.19; N, 3.49.

*N'-Benzylidene-3-(6,11-dioxo-6,11-dihydro-12H-benzo[b]phenoxazin-12-yl)butanehydrazide* (**12d**) was synthesized from **5d** to afford 0.13 g (84%) of **12d**; m.p.: 154 °C (decomp.); R_f_ = 0.74 (acetone–hexane, 1:1); IR (KBr), *ν*, cm^−1^: 3230 (CONH), 1693, 1668, 1635 (CO); ^1^H-NMR (400 MHz, DMSO-*d*_6_): δ = 1.60 (d, (0.6)3H (*E*), *J* = 6.9 Hz, CH_3_), 1.69 (d, (2.4)3H (*Z*), *J* = 7.0 Hz, CH_3_), 2.91 (d, 1H, *J* = 15.7 Hz, CH_2_), 3.57 (d, 1H, *J* = 15.7 Hz, CH_2_), 4.35–4.56 (m, 1H, *CH*CH_2_), 6.74–8.21 (m, 13H, H_aromatic_), 8.17 (s, 1H, CH), 11.29 (s, (0.7)1H (*Z*), NHN), 11.33 (s, (0.3)1H (*E)*, NHN); ^13^C-NMR (100 MHz, DMSO-*d*_6_): δ = 20.63 (CH_3_ (*E*)), 21.39 (CH_3_ (*E*)), 37.28 (CH_2_), 57.42 (CH), 116.20, 120.36, 120.65, 124.95, 125.17, 126.04, 127.01, 128.54, 128.60, 129.49, 129.72, 130.36, 131.13, 132.56, 133.55, 133.82, 134.47, 136.77, 145.88 (2C+ C_aromatic_), 142.81 (CN (*Z*)), 148.66 (CN (*E*)), 172.07, 175.98, 181.21 (3CO); HRMS calculated for C_27_H_21_N_3_O_4_ [M+H]^+^ 452.1532 found 452.1601; Anal. Calcd. (%) for: C, 71.83; H, 4.69; N, 9.31, found: C, 71.70; H, 4.28; N, 9.51.

*N-(2,5-Dimethyl-1H-pyrrol-1-yl)-3-(6,11-dioxo-6,11-dihydro-12H-benzo[b]phenoxazin-12-yl)butanamide* (**13**) was synthesized from **6** to afford 0.3 g (43%) of **13**; m.p.: 185 °C (decomp.); R_f_ = 0.69 (acetone–hexane, 1:1); IR (KBr), *ν*, cm^−1^: 3250 (CONH), 1704, 1666, 1644 (CO); ^1^H-NMR (400 MHz, Acetone-*d*_6_): δ = 1.65 (d, 3H, *J* = 6.9 Hz, CH_3_), 1.99 (s, 6H, 2CH_3_), 3.18 (dd, 2H, *J* = 7.3, 15.7 Hz, CH_2_), 4.44–4.84 (m, 1H, *CH*CH_2_), 5.58 (s, 2H, 2CH), 6.81–7.13, 7.79–8.05 (m, 8H, H_aromatic_), 9.91 (d, 1H, *J* = 16.4 Hz, NHN); ^13^C-NMR (100 MHz, Acetone-*d*_6_): δ = 11.31 (2 CH_3_), 21.28 (CH_3_), 39.85 (CH_2_), 57.99 (CH), 103.97 (2CH), 117.37, 121.44, 125.37, 125.98, 126.20, 127.01, 131.49, 131.83, 132.45, 133.50, 134.46, 134.77, 136.83, 137.12 (2C+2C+C_aromatic_), 170.28, 174.03, 181.92 (3CO); HRMS calculated for C_26_H_23_N_3_O_4_ [M+H]^+^ 442.1689 found 442.1761; Anal. Calcd. (%) for: C, 70.74; H, 5.25; N, 9.52, found: C, 70.60; H, 5.38; N, 9.41.

#### 3.2.7. General Procedure for Synthesis of *N'*-Substituted-3-(6,11-dioxo-6,11-dihydro-12*H*-benzo[*b*]phenoxazin-12-yl)butanehydrazides **12e–g**

A mixture of the compound **5e–g** (0.35 mmol), 2,3-dichloro-1,4-naphthoquinone (0.08 g, 0.35 mmol), sodium acetate (0.14 g, 1.75 mmol), and acetone (20 mL) was heated under reflux for 14 h. The liquid fraction was removed under reduced pressure and the target product was isolated by column chromatography (acetone–hexane, 1:1).

*3-(6,11-Dioxo-6,11-dihydro-12H-benzo[b]phenoxazin-12-yl)-N'-(4-methoxybenzylidene)butanehydrazide* (**12e**). Yield 0.10 g (35.5%); m.p.: 146–147 °C; R_f_ = 0.60 (acetone–hexane, 1:1); IR (KBr), *ν*, cm^−1^: 2924 (CONH), 2852 (OCH_3_), 1667, 1625, 1604 (CO); ^1^H-NMR (400 MHz, DMSO-*d*_6_): δ = 1.53 (d, (0.6)3H (*E*), *J* = 6.9 Hz, CH_3_), 1.62 (d, (2.4)3H (*Z*), *J* = 7.0 Hz, CH_3_), 2.76–2.92 (m, 1H, CH_2_), 3.43 (dd, 1H, *J* = 7.9, 15.5 Hz, CH_2_), 3.77 (s, 3H, OCH_3_), 4.22–4.38 (m, 1H, *CH*CH_2_), 7.83–7.97 (m, 13H, H_aromatic_+CH), 11.25 (s, (0.7)1H (*Z*), NHN), 11.27 (s, (0.3)1H (*E)*, NHN); ^13^C-NMR (100 MHz, DMSO-*d*_6_): δ = 20.99 (CH_3_ (*E*)), 21.68 (CH_3_ (*Z*)), 40.19 (CH_2_), 55.28 (CH), 57.22 (OCH_3_), 114.12, 114.26, 116.24, 119.91, 126.06, 126.61, 128.15, 128.62, 130.00, 130.78, 132.17, 134.05, 136.48, 138.80, 145.14, 146.11, 160.48 (2C+ C_aromatic_), 142.73 (CN (*Z*)), 148.14 (CN (*E*)), 171.73, 176.02, 181.12 (3CO); HRMS calculated for C_28_H_23_N_3_O_5_ [M+H]^+^ 482.1634 found 482.1708; Anal. Calcd. (%) for: C, 69.84; H, 4.81; N, 8.73, found: C, 69.71; H, 4.58; N, 8.81.

*3-(6,11-Dioxo-6,11-dihydro-12H-benzo[b]phenoxazin-12-yl)-N'-(furan-2-ylmethylene)butanehydrazide* (**12f**). Yield 0.11 g (73%); m.p.: 69–70 °C; R_f_ = 0.58 (acetone–hexane, 1:1); IR (KBr), *ν*, cm^−1^: 2924 (CONH), 1722, 1673, 1629 (CO); ^1^H-NMR (400 MHz, DMSO-*d*_6_): δ = 1.53 (d, (1)3H (*E*), *J* = 6.8 Hz, CH_3_), 1.61 (d, (2)3H (*Z*), *J* = 6.9 Hz, CH_3_), 2.64 (dd, 1H, *J* = 7.6, 15.6 CH_2_), 2.82 (dd, 1H, *J* = 6.8, 16.3 Hz, CH_2_), 4.27 (dd, (0.5)1H, *J* = 7.1, 14.0 Hz, *CH*CH_2_), 4.35 (dd, (0.5)1H, *J* = 7.0, 14.1 Hz, *CH*CH_2_), 6.54–7.06, 7.68–8.09 (9H, m, H_aromatic_+3CH), 11.28 (s, (0.7)1H (*Z*), NHN), 11.33 (s, (0.3)1H (*E)*, NHN); HRMS calculated for C_25_H_19_N_3_O_5_ [M+H]^+^ 442.1325 found 442.1707; Anal. Calcd. (%) for: C, 68.02; H, 4.34; N, 9.52, found: C, 68.50; H, 4.48; N, 9.62.

*3-(6,11-Dioxo-6,11-dihydro-12H-benzo[b]phenoxazin-12-yl)-N'-(thiophen-2-ylmethylene)butanehydrazide* (**12g**). Yield 0.06 g (40%); m.p.: 77–78 °C; R_f_ = 0.61 (acetone–hexane, 1:1); IR (KBr), *ν*, cm^−1^: 3069 (CONH), 1663, 1664, 1625 (CO); ^1^H-NMR (400 MHz, DMSO-*d*_6_): δ = 1.52 (d, (0.6)3H (*E*), *J* = 6.9 Hz, CH_3_), 1.60 (d, (2.4)3H (*Z*), *J* = 7.0 Hz, CH_3_), 2.82 (dd, 1H, *J* = 8.2, 15.1 CH_2_), 2.82 (dd, 1H, *J* = 7.3, 15.7 Hz, CH_2_), 4.24–4.33 (m, 1H, *CH*CH_2_), 6.83–8.09 (m, 11H, H_aromatic_+3CH), 8.28 (s, 1H, CH), 11.35 (s, (0.7)1H (*Z*), NHN), 11.36 (s, (0.3)1H (*E)*, NHN); ^13^C-NMR (100 MHz, DMSO-*d*_6_): δ = 22.04 (CH_3_ (*E*)), 24.37 (CH_3_ (*Z*)), 30.06 (CH_2_), 58.81 (CH), 111.33, 116.36, 120.75, 124.43, 128.21, 128.24, 130.47, 131.29, 134.49, 136.62, 137.90, 139.56, 141.22, 148.89, (2C+3C+C_aromatic_), 125.65 (CN (*E*)), 126.53 (CN (*Z*)), 163.01 (C*C*S), 172.08, 176.07, 181.73 (3CO); HRMS calculated for C_25_H_19_N_3_O_4_S [M+H]^+^ 458.1634 found 458.1166; Anal. Calcd. (%) for: C, 65.63; H, 4.19; N, 9.18, found: C, 65.74; H, 4.28; N, 9.41.

### 3.3. Biology

#### 3.3.1. Diffusion Technique

Antimicrobial activity of compounds has been evaluated by diffusion in peptone on solid nutrient medium (nutrient agar—for bacteria, wort agar—for fungi). The microbial loading was 10^9^ cells/mL. The duration of incubation for bacteria was 24 h at 35 °C and for fungi it was 48–72 h at 28–30 °C. The results were recorded by measuring the zones surrounding the disk. Control disk contained vancomycin (for bacteria) or nystatin (for fungi) as a standard substance.

#### 3.3.2. Serial Dilution Technique

Compounds were tested according to standard microbroth dilution for determination of minimum inhibitory concentration (MIC). The certain volume of solution of compound in DMSO was brought in nutrient medium (nutrient meat-extract—for bacteria, wort—for fungi). The tested compounds were dissolved in DMSO and the concentration range was 500–1.9 μg/mL. The inoculum of bacteria and fungi was inoculated in nutrient medium. The duration of incubation of bacteria was 24–72 h at 37 °C for bacteria and 30 °C for fungi. The results were estimated according to the presence or absence of growth of microorganisms.

## 4. Conclusions

A series of compounds containing hydrazide, pyrrole and chloroquinoxaline moieties were synthesized from 3-[(2-hydroxyphenyl)amino]butanoic and 3-[(2-hydroxy-5-methyl(chloro)phenyl)amino]butanoic acids. The subsequent reactions of the obtained compounds with 2,3-dichloro-1,4-naphthoquinone provided respective benzo[*b*]phenoxazine derivatives. The screening of antimicrobial and antifungal activity of the synthesized compounds has revealed that benzo[*b*]phenoxazine derivatives **9a–c**, **10c**, and **12f** are active against Gram-positive bacteria *S. aureus* and *M. luteum* at lower concentrations. Among the most active antifungal compounds, carboxylic acids **9a,c** can be mentioned. Thiophene derivative **12g**, which MIC value against *A. niger* was as low as 3.9 µg/mL, showed better inhibiting action than antifungal agent nystatin.

## References

[1] Rice L.B. (2006). Unmet medical needs in antibacterial therapy. Biochem. Pharmacol..

[2] Patočka J. (2011). β-Amino acids and their natural biologically active derivatives. 5. Derivatives of unusual alicyclic and heterocyclic β-amino acids. Mil. Med. Sci. Lett..

[3] Chemin L.S., Buisine E., Yardley V., Kohler S., Debreu M.A., Landry V., Sergheraert C., Croft S.L., Siegel R.L.K., Charvet E.D. (2001). 2- and 3-Substituted 1,4-naphthoquinone derivatives as subversive substrates of trypanothione reductase and lipoamide dehydrogenase from *Trypanosoma cruzi*: Synthesis and correlation between redox cycling activities and *in vitro* cytotoxicity. J. Med. Chem..

[4] Uchimiya M., Stone A.T. (2009). Reversible redox chemistry of quinones: Impact on biogeochemical cycles. Chemosphere.

[5] Pérez-Sacau E., Díaz-Peñate R.G., Estévez-Braun A., Ravelo A.G., García-Castellano J.M., Pardo L., Campillo M. (2007). Synthesis and pharmacophore modeling of naphthoquinone derivatives with cytotoxic activity in human promyelocytic leukemia HL-60 cell line. J. Med. Chem..

[6] Tandon V.K., Chhor R.B., Singh R.V., Rai S., Yadav D.B. (2004). Design, synthesis and evaluation of novel 1,4-naphthoquinone derivatives as antifungal and anticancer agents. Bioorg. Med. Chem. Lett..

[7] Sasaki K., Abe H., Yoshizaki F. (2002). *In vitro* antifungal activity of naphthoquinone derivatives. Biol. Pharm. Bull..

[8] Tandon V.K., Maurya H.K., Tripathi A., ShivaKesva G.B., Shukla P.K., Srivastava A., Panda D. (2009). 2,3-Disubstituted-1,4-naphthoquinones, 12*H*-benzo[*b*]phenothiazine-6,11-diones and related compounds: Synthesis and biological evaluation as potential antiproliferative and antifungal agents. Eur. J. Med. Chem..

[9] Voskienė A., Sapijanskaitė B., Mickevičius V., Kantminienė K., Stasevych M., Komarovska-Porokhnyavets O., Musyanovych R., Novikov V. (2011). Synthesis, chemical properties and antimicrobial activity of 2- and 2,3-substituted[(tetrahydro-2,4-dioxopyrimidin-1(2*H*)-yl)-phenoxy]naphthalene-1,4-diones. Monatsh. Chem..

[10] Voskienė A., Sapijanskaitė B., Mickevičius V., Jonuškienė I., Stasevych M., Komarovska-Porokhnyavets O., Musyanovych R., Novikov V. (2012). Synthesis and microbial evaluation of new 2- and 2,3-diphenoxysubstituted naphthalene-1,4-diones with 5-oxypyrrolidine moieties. Molecules.

[11] Anusevičius K., Jonuškienė I., Mickevičius V. (2013). Synthesis and antimicrobial activity of *N*-(4-chlorophenyl)-β-alanine derivatives with an azole moiety. Monatsh. Chem..

[12] Ibis C., Tuyun A.F., Bahar H., Ayla S.S., Stasevych M.V., Musyanovych R.Y., Komarovska-Porokhnyavets O., Novikov V. (2013). Synthesis of novel 1,4-naphthoquinone derivatives: Antibacterial and antifungal agents. Med. Chem. Res..

[13] Jordão A.K., Novais J., Leal B., Escobar A.C., dos Santos H.M., Castro H.C., Ferreira V.F. (2013). Synthesis using microwave irradiation and antibacterial evaluation of new *N*,*O*-acetals and *N*,*S*-acetals derived from 2-amino-1,4-naphthoquinones. Eur. J. Med. Chem..

[14] Tandon V.K., Yadav D.B., Singh R.V., Vaish M., Chaturvedi A.K., Shukla P.K. (2005). Synthesis and biological evaluation of novel 1,4-naphthoquinone derivatives as antibacterial and antiviral agents. Bioorg. Med. Chem. Lett..

[15] Bhasin D., Chettiar S.N., Etter J.P., Mok M., Li P.-K. (2013). Anticancer activity and SAR studies of substituted 1,4-naphthoquinones. Bioorg. Med. Chem..

[16] Silva T.M.S., Camara C.S., Barbosa T.P., Soares A.Z., Cunha L.C., Pinto A.C., Vargas M.D. (2009). Molluscicidal activity of synthetic lapachol amino and hydrogenated derivatives. Bioorg. Med. Chem..

[17] Lee K., Cho S.H., Lee J.H., Goo J., Lee S.Y., Boovanahalli S.K., Yeo S.K., Lee S.J., Kim Y.K., Kim D.H. (2013). Synthesis of a novel series of 2-alkylthio substituted naphthoquinones as potent acyl-CoA: Cholesterol acyltransferase (ACAT) inhibitors. Eur. J. Med. Chem..

[18] Ali M.M., Ismail M.M.F., El-Gaby M.S.A., Zahran M.A., Ammar Y.A. (2000). Synthesis and antimicrobial activities of some novel quinoxalinone derivatives. Molecules.

[19] Badran M.M., Moneer A.A., Refaat H.M., El-Malah A.A. (2007). Synthesis and antimicrobial activity of novel quinoxaline derivatives. J. Chin. Chem. Soc..

[20] González M., Cerecetto H. (2012). Quinoxaline derivatives: A patent review (2006-present). Exp. Opin. Ther. Pat..

[21] Kumar A., Verma A., Chawla G. (2009). Synthesis, antiinflammatory and antimicrobial activities of new hydrazone and quinoxaline derivatives. Int. J. ChemTech Res..

[22] Patidar A.K., Jeyakandan M., Mobiya A.K., Selvam G. (2011). Exploring potential of quinoxaline moiety. Int. J. PharmTech Res..

[23] Wu P., Su Y., Liu X., Yan J., Ye Y., Zhang L., Xu J., Weng S., Li Y., Liu T. (2012). Discovery of novel morpholino-quinoxalines as PI3Kα inhibitors by pharmacophore-based screening. Med. Chem. Commun..

[24] Noolvi M.N., Patel H.M., Bhardwaj V., Chauhan A. (2011). Synthesis and *in vitro* antitumor activity of substituted quinazoline and quinoxaline derivatives: Search for anticancer agent. Eur. J. Med. Chem..

[25] Mielcke T.R., Mascarello A., Fillipi-Chiela E., Zanin R.F., Lenz G., Leal P.C., Chiaradia L.D., Yunes R.A., Nunes R.J., Battastini A.M. (2012). Activity of novel quinoxaline-derived chalcones on *in vitro* glioma cell proliferation. Eur. J. Med. Chem..

[26] Fraga C.A.M., Barreiro E. (2006). Medicinal chemistry of *N*-acylhydrazones: New lead-compounds of analgesic, antiinflammatory and antithrombotic drugs. J. Curr. Med. Chem..

[27] Rollas S., Küçükgüzel S.G. (2007). Biological activities of hydrazone derivatives. Molecules.

[28] Zulkepli N.A., Rou K.V.K., Sulaiman W.N.H.W., Salhin A., Saad B., Seeni A. (2011). A synthetic hydrazone derivative acts as an apoptotic inducer with chemopreventive activity on a tongue cancer cell line. Asian Pac. J. Cancer Prev..

[29] Wardakhan W.W., El-Sayed N.N.R., Mohareb M. (2013). Synthesis and anti-tumor evaluation of novel hydrazide and hydrazide-hydrazone derivatives. Acta Pharm..

[30] Mickevičius V., Voskienė A., Jonuškienė I., Kolosej R., Šiugždaitė J., Venskutonis P.R., Kazernavičiūtė R., Brazienė Z., Jakienė E. (2013). Synthesis and biological activity of 3-[phenyl(1,3-thiazol-2-yl)-amino]propanoic acids and their derivatives. Molecules.

[31] Tumosienė I., Jakienė E., Kantminienė K., Rutkauskas K., Beresnevičius Z.J. (2010). Synthesis and plant growth regulating activity of halo derivatives of 3,3'-(arylimino)dipropanoic acids. CHEMIJA.

[32] Brokaitė K., Mickevičius V., Mikulskienė G. (2006). Synthesis and structural investigation of some 1,4-disubstituted 2-pyrrolidinones. ARKIVOC.

[33] Anusevicius K., Mickevicius V., Stasevych M., Zvarych V., Komarovska-Porokhnyavets O., Novikov V., Tarasova O., Gloriozova T., Poroikov V. Synthesis and chemoinformatics analysis of *N*-aryl-*β*-alanine derivatives. Res. Chem. Intermed..

[34] Stankevičienė R., Jonuškienė I., Baranauskaitė R., Mickevičius V. (2010). The influence of *N*-(2-hydroxyphenyl)-β-alanines and products of their interaction with 2,3-dichloro-1,4-naphthoquinone on barley (*Hordeum vulgare* L.) growth and flavonoids formation. Chem. Technol..

[35] CLSI (2015). Performance Standards for Antimicrobial Disk Susceptibility Tests.

[36] CLSI (2008). Reference Method for Broth Dilution Antifungal Susceptibility Testing of Filamentous Fungi.

[37] Mickevičius V., Baltrušis R., Beresnevičius Z. (1991). Synthesis and cyclization of *N*-(2-hydroxyphenyl)-β-alanines and *N*-(2-benzylhydroxyphenyl)-β-alanines. Khim. Geterotsikl. Soedin..

